# Urinary catheterization from 1997 to 2018: a Dutch population-based cohort

**DOI:** 10.1177/17562872211007625

**Published:** 2021-04-12

**Authors:** Sophie A. Berendsen, Tess van Doorn, Bertil F. M. Blok

**Affiliations:** Department of Urology, Erasmus MC, Dr. Molewaterplein 40, Rotterdam 3015 GD, The Netherlands; Department of Urology, Erasmus Medical Center, Rotterdam, The Netherlands; Department of Urology, Erasmus Medical Center, Rotterdam, The Netherlands

**Keywords:** external catheterization, health expenditures, indwelling catheterization, intermittent urethral catheterization, prevalence, urinary catheterization

## Abstract

**Background:**

Our aim was to evaluate the use of indwelling, intermittent and external urinary catheters in neurogenic and non-neurogenic bladder patients in the Netherlands from 1997 to 2018.

**Methods:**

Data were retrieved from a population-based cohort containing information about the extramural use of medical devices in the insured population in the Netherlands. The insured population increased from 9.9 million people in 1997 to 17.1 million people in 2018 (64–100% of the Dutch population). Users are expressed by users per 100,000 insured people and total users, corrected for the overall Dutch population. The expenditures are corrected for inflation and expressed by total costs and costs per user.

**Results:**

During this 21-year period, indwelling catheter (IC) users doubled from 159 per 100,000 people (24,734 users) to 315 per 100,000 people (54,106 users). Clean intermittent catheter (CIC) users increased from 92 per 100,000 people (14,258 users) in 1997 to 267 per 100,000 people (45,909 users) in 2018. Of all users, 20.7% had an associated neurogenic disorder and 44.9% a non-neurogenic disorder in 2018. The total expenditure on extramural use of urinary catheters increased from 27.7 million euros in 1997 to 84.4 million euros in 2018. IC costs increased from 6.0 million euros in 1997 to 6.7 million euros in 2018, while CIC costs rose from 16.4 million euros to 74.6 million euros. Urine drainage bag costs decreased from 17.2 million in 2001 to 5.3 million in 2018.

**Conclusions:**

IC use has increased substantially over the past 21 years, despite the fact that CIC use increased as well. It seems that the main driver behind the prevalence in IC and CIC use, is the rise in incontinence care in older patients and the adaption of preferred CIC use in professional guidelines. At least one fifth of all users catheterize due to neurogenic reasons.

## Introduction

Indwelling catheterization has been used for centuries for those who fail to drain their bladder (urinary retention) or fail to control their bladder (urinary incontinence). Urinary retention is seen in patients with neurogenic and non-neurogenic disorders. The most common associated neurogenic disorders are spinal cord injury, spina bifida or meningomyelocele, multiple sclerosis and morbus Parkinson.^1^ Associated non-neurogenic disorders are, for example, benign prostate obstruction, or the onset of urinary retention can occur post-partum or post-pelvic surgery.^2^ In many cases, a single underlying cause is unknown, and underactivity of the detrusor muscle might be a contributing factor. Unfortunately, it is often unclear how detrusor underactivity occurs and is therefore referred to as idiopathic.

Another reason for catheterization is urinary incontinence, which can be subdivided into stress, urge, and mixed urinary incontinence. Different treatment methods are available for urinary incontinence, but pads are widely used, especially in nursing homes with mostly vulnerable patient populations. The risk of pressure sores is high in immobile and fragile people using pads.^3^ Therefore, together with the high workload due to incontinence, pads are often replaced for indwelling catheters (ICs) to prevent, or after the development of, pressure sores. In patients with urinary incontinence treated with an IC, urine follows the path of least resistance and is drained by the IC, preventing any leakage. Another treatment option is the use of a condom catheter. This catheter, also referred as an external catheter (EXC), is not inserted into the bladder, but is fitted around the penis with a sheath similar to a condom. It is used for urinary incontinence and urinary retention, but is not advised for patients with neurogenic high-pressure bladders due to the elevated risk for renal deterioration.^4,5^

For patients suffering from only urinary retention, the treatment of choice is clean intermittent catheterization (CIC). CIC is the insertion and removal of a catheter in the bladder to drain the urine for a short time and is mostly performed four to six times a day. Compared with indwelling catheterization, CIC is the preferred treatment due to the reduced risk for complications like catheter-associated urinary tract infections, bladder stones, and renal deterioration.^4,6^ However, the ability to perform CIC depends on different factors, including personal factors (e.g. hand function and position of the urethral meatus) and healthcare policies for the reimbursement of urinary catheters.

According to the review of Feneley *et al*.^7^ the costs of ICs accounted for around 380 million USD in 2007. Looking at the economic burden of all urinary catheters, the market size was valued at 3.4 billion USD in 2015, with a gradual growth in future perspective. Around 60% of the expenditure on urinary catheters is consumed by disposable intermittent urinary catheters.^8^ In The Netherlands, urinary equipment, including ICs and disposable catheters for CIC, is reimbursed for neurogenic and non-neurogenic bladder patients. It is hypothesized that the prevalence of CIC use is higher in countries with reimbursement agreements than countries without or with partial reimbursement, but actual data on urinary catheter use are lacking for most countries. A previous study showed that CIC use and costs in The Netherlands increased substantially in the past 2 decades.^9^ This increase in CIC use is in line with the overall population growth, the aging population, and the adaption of the recommendation in professional guidelines of urologist and rehabilitation physicians for the preferred use of intermittent catheters.

The present study investigated the trends in extramural (non-hospitalized and non-institutionalized) use of all urinary catheters, including indwelling and external catheters in neurogenic and non-neurogenic patients, including the costs in the Netherlands between 1997 and 2018. In addition, we examined the age and sex distribution for IC, CIC and EXC use. This study was performed in comparison with the results of a previous study on intermittent urinary catheter use in the Netherlands between 1997 and 2018.^9^ Our hypothesis was that the adaption of the recommendation of CIC use for urinary retention resulted in a decline of IC and EXC use in the Netherlands. Furthermore, we hypothesized a large amount of the neurogenic bladder patients use intermittent catheters for their treatment of chronic urinary retention, and that the prevalence of IC and EXC use increases with age.

## Methods

### Study design

For this retrospective, population-based database study, data were obtained from the Drug and Medical Devices Information System (Genees-en hulpmiddelen Informatie Project; GIP) of the Dutch Health Care Institute (Zorginstituut Nederland). This database contains information about all reimbursed prescriptions on extramural (non-hospitalized and non-institutionalized) medication and medical devices. Between 1997 and 2005, the GIP database only contains information about insured people under the Public Health Insurance Law (Ziekenfondswet), which was 63% of the total Dutch population. After 2006, the Healthcare Insurance Act was implemented and all insured people were included in the GIP database (100% of the total Dutch population in 2018). Data are based on number of prescriptions per patient per year. All data used were obtained and handled according to the Dutch privacy laws.

The following time trends were evaluated for the use of urinary catheters per year:

(1) number of ICs, intermittent catheter and external catheter users in the Dutch population between from 1997 to 2018;
(2) the distribution of different type of catheter users among different sex and age groups from 1997 to 2018;
(3) the distribution of different type of catheter users;
(4) among neurogenic and non-neurogenic bladder patients from 2012 to 2018;
(5) costs for the total population of indwelling, intermittent and external catheters used from 1997 to 2018;
(6) costs of different urinary catheter types and accessories per individual catheter user from 1997 to 2018.

In the Netherlands, all declarations of medical devices by pharmacists or medical devices suppliers are coded through ZI-numbers or the Generic Product Code for devices (Generieke Productcodes Hulpmiddelen: GPH). The ZI-numbers are published in the G-Standaard by Z-index, a database containing product information of medicines and medical devices that are dispensed or used in the Dutch healthcare system.^10^ The GPH-codes are managed by Vektis,^11^ a non-commercial database responsible for relaying pseudonymised data from the healthcare insurers to the National Healthcare Institute. The health insurance companies share these declarations with the GIP database. The GIP database links the ZI-numbers and GPH-codes to a corresponding ISO9999-code, which is translated into a classification. All urinary catheters and accessories are classified under the monitor code ‘A1535 catheters’ and are subcategorized by different ISO-codes. For this study, data were obtained from different ISO-codes, including the ISO-codes for ICs, disposable intermittent catheters, external catheters, and urine drainage bags and other accessories (e.g. disposable intermittent catheters is ISO92406).

### Data analysis

Before data were obtained for the different type of ISO-codes, all links between ZI-numbers/GPH-codes and ISO-codes were analyzed and checked by visual control of the product names. In addition, all occurring ZI-numbers were checked with the product information in BeverOnline, a medical device database from Nigella IT .^12^ Some improper links between the ZI-numbers, GPH-codes and ISO-codes or incorrectly classified products were removed. This included an improved classification for medical devices which had been incorrected classified. All individual catheter users were linked with a unique pseudonymized number to specific ISO-codes. After the reclassification of the different medical devices, we categorized all intermittent and IC users for neurogenic or non-neurogenic causes. For this classification we used data of specific combined diagnosis and treatment codes (DBC code or Diagnose Behandeling Combinatie code) in the Netherlands from 2012 to 2018. The modified DBC code was introduced on 1 January 2012 and is a code based on diagnosis and treatment for every individual patient. There are no reliable DBC codes available before 2012. In the Netherlands, hospitals are reimbursed for patients based on the DBC, and all DBC codes are maintained in the DBC-information system by the Dutch Healthcare Authority (Nederlandse Zorgautoriteit; NZA). A predefined list of different DBC codes was made, and every DBC code was labeled for neurogenic or non-neurogenic cause (appendix A). Every intermittent and IC user with a DBC code from the predefined list from 2012 to 2018 was categorized into a neurogenic or non-neurogenic cause. If patients had multiple DBC codes, the neurogenic DBC overruled the non-neurogenic DBC. We adjusted the development of expenditures of intermittent disposable catheters for inflation using the Consumer Price Index (CPI) published by Statistics Netherlands.^13^ By adjusting for the general price development of consumer goods and services, the changes in the expenditures on intermittent disposable catheters and accessories are the result of changes in volume and specific price movements. All expenditures are expressed in prices of 2018 in euros.

## Results

The GIP database contains information about the insured population in the Netherlands. In 2006, the Dutch Healthcare Insurance Act was implemented. The insured population increased from 16.2 million people (99% of the Dutch population) in 2006 to 17.1 million people (100% of the Dutch population) in 2018. Prior to 2006, data were only available for people insured under the Ziekenfondswet, which increased from 9.9 million individuals (64% of the Dutch population) in 1997 to 10.2 million (63% of the Dutch population) in 2005. The remaining population was insured by private healthcare insurers and not included in the GIP database. The prevalence of extramural urinary catheter users is expressed by users per 100,000 people. If stratified for age and sex, users are expressed by 100,000 people in the same specific age and sex. Total number of catheter users was calculated using population data over the years 1997–2018 of Statistic Netherlands (Centraal Bureau voor de Statistiek).^14^

### Catheter users

**Figure 1.**
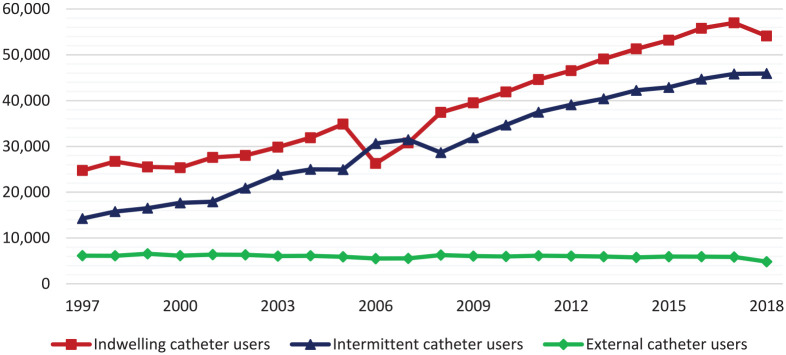
Number of catheter users corrected for the total population from 1997 to 2018.

The number of all catheter users increased from 291 users per 100,000 people in 1997 to 610 users per 100,000 people in 2018. IC users doubled from 159 users per 100,000 people in 1997 to 315 per 100,000 people in 2018. In absolute numbers, IC users increased from 24,734 to 54,106 users. In the same 21-year period, the number of CIC users almost tripled from 92 per 100,000 people to 267 per 100,000 people. Absolute CIC users increased from 14,258 to 45,909 users.^9^ Of all IC and CIC users, mixed use was found in 16 per 100,000 in 1997 (2499 absolute users) to 28 per 100,000 people in 2018 (4791 absolute users). A decrease was seen in EXC use, from 40 users per 100,000 people in 1997 to 28 users per 100,000 people in 2018. Absolute EXC users decreased from 6158 to 4841 users. Total users for indwelling, intermittent, and external catheters are shown in Figure 1.

Table 1 presents the characteristics of the different type of catheter users for age and sex. User numbers are expressed in users per specific age category compared with 100,000 people in the same specific age category. The use of ICs only declined in users under the age of 25-years old. Female IC users between 65 and 74 years increased with 85%, from 91 per 100,000 people in 1997 to 355 per 100,000 people in 2018. Male IC users above 85-years old increased from 4704 per 100,000 people in 1997 to 8133 per 100,000 people in 2018, an increase of 73%. The highest increase of all catheter use, was seen in male CIC users between 75 to 85-years old, from 453 users per 100,000 people in 1997 to 1700 users per 100,000 people in 2018 (275%). External catheter users decreased in every age category. What is interesting in Table 1, is the increased risk of catheterization by higher age. A total of 0.8% of 65–75-year-old men used at least one IC in 2018. In the same year, IC was used in 8.1% for men over 85-years old.

**Table 1.** Characteristics of catheter users in 1997 and 2018.

Characteristics	Indwelling catheters			Intermittent catheters			External catheters		
	1997	2018	Change (%)	1997	2018	Change (%)	1997	2018	Change (%)
Total users	159	315	98	92	267	192	40	28	−29
Sex and age distribution									
Male users	180	396	119	92	334	262	85	57	−33
0–25 years	11	6	−46	25	39	54	6	4	−23
25–45 years	21	24	13	41	84	107	35	14	−59
45–65 years	122	159	30	111	272	145	73	37	−49
65–75 years	493	839	70	287	952	232	191	125	−35
75–85 years	1564	2464	58	453	1700	275	660	312	−53
85+ years	4704	8133	73	719	2040	184	1544	743	−52
Female users	140	235	68	91	201	121			
0–25 years	7	5	−23	35	42	21			
25–45 years	26	34	33	60	113	88			
45–65 years	85	128	50	102	212	109			
65–75 years	191	355	85	157	392	151			
75–85 years	603	886	47	221	589	167			
85+ years	2271	3210	41	441	724	64			

Users are expressed by users per 100,000 insured people in that specific age and sex category for the total Dutch population.

### Etiology

Of all 95,234 catheter users in 2018, 19,680 users (20.7%) had a neurogenic cause as underlying disease. A total of 42,749 users (44.9%) were classified as non-neurogenic. The remaining 32,806 users (34.4%) had no registered DBC after 2012 and the underlying condition could not be defined (Table 2). IC users had a neurogenic cause in 8904 (18.1%) cases, in 20,407 cases a non-neurogenic cause, and the remaining 20,004 (40.6%) was undefined. A total of 9579 (23.3%) CIC users had a neurogenic cause, 20,070 (48.8%) had a non-neurogenic cause, and in 11,480 CIC users (27.9%) a cause could not be defined. Of all mixed users, 1197 (25%) had a neurogenic cause, 2272 (47.4%) had a non-neurogenic cause, and the remaining 1322 (27.6%) was undefined as well.

**Table 2.** Etiology of catheterization in number of users for the total population.

	Indwelling catheters	Intermittent catheters	Intermittent and indwelling catheters	Total
	Users (%)	Users (%)	Users (%)	Users (%)
Neurogenic	8904 (18.1)	9579 (23.3)	1197 (25.0)	19,680 (20.7)
Non-neurogenic	20,407 (41.4)	20,070 (48.8)	2272 (47.4)	42,749 (44.9)
Unknown^a^	20,004 (40.6)	11,480 (27.9)	1322 (27.6)	32,806 (34.4)
Total number	49,314 (100)	41,129 (100)	4791 (100)	95,234 (100)

a Users without recent DBC registration.

DBC, Diagnose Behandeling Combinatie.

Of all neurogenic catheter users, 45.2% (8904 cases) used only ICs, 48.7% (9579 cases) used only intermittent catheters, and 6.1% used both types of catheters.

### Costs of catheter users

**Figure 2.**
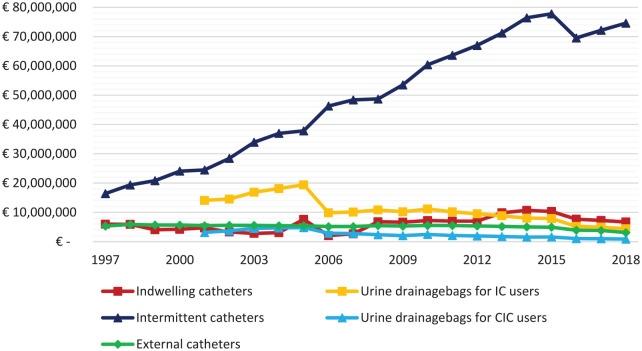
Number of costs for the total population, corrected for inflation. Costs for urine drainage bags are available since 2001. CIC, clean intermittent catheter; IC, indwelling catheter.

All costs are corrected for inflation and expressed in euros of 2018. Detailed information about the use on urine drainage bags for IC and CIC users was only available after 2001. Total expenditure on extramural use of urinary catheters increased from 27.7 million euros in 1997 to 84.4 million euros in 2018. The total costs for urine drainage bags decreased from 17.2 million in 2001 to 5.3 million in 2018. All urinary catheters and urine drainage bags costed 89.7 million euro in 2018. The total costs for the different type of catheters are shown in Figure 2. For IC users, costs increased from 6.0 million euros in 1997 to 6.7 million euros in 2018. Costs for urine drainage bags for IC users decreased from 14.1 million euros in 2001 to 4.4 million euros in 2018. Costs for CIC users increased from 16.4 million euros in 1997 to 74.6 million euros in 2018. Costs for urine drainage bags for CIC users decreased from 3.1 million euros in 2001 to 0.9 million euros in 2018. Total costs for external catheter users decreased from 5.3 million euros in 1997 to 3.1 million euros in 2018.

**Figure 3.**
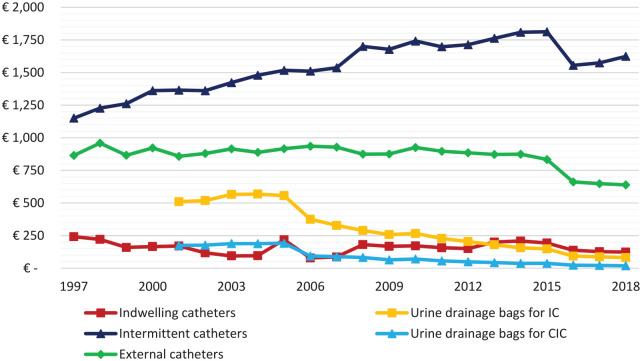
Annual costs per user, corrected for inflation. Annual costs for urine drainage bags are available since 2001. CIC, clean intermittent catheter; IC, indwelling catheter.

Costs per user are shown in Figure 3. Annual costs per IC user decreased from 242 euros in 1997 to 124 euros in 2018. Urine drainage bags for IC users decreased from 510 euros in 2001 to 81 euros in 2018. Annual costs per CIC user increased from 1151 euros in 1997 to 1624 euros in 2018, urine drainage bags for CIC users decreased from 175 euros in 2001 to 19 euros in 2018. Annual costs per external catheter user decreased in the same time period from 865 euros to 639 euros.

## Discussion

Urinary catheters are a widely used therapy for patients with an impaired bladder function. This study explored the trends in extramural use and costs of indwelling and external catheters in comparison with the results of intermittent urinary catheter use in the Netherlands from 1997 to 2018. The findings clearly show an increase in absolute and relative urinary catheter use and costs. In the past 2 decades, IC use more than doubled, alongside the threefold increase in CIC use. These results were rather unexpected, as we hypothesized that IC use would decline if CIC use increased. A possible explanation is the increased prevalence of urinary incontinence due to an aging population. In the United Kingdom, around 1.3 million people sought help for incontinence in 2006–2007, but this number rose to 2.3 million people in 2010–2011. The risk of incontinence increases with age; 14% of 64–69-year-old people have urinary incontinence, which increases to 45% for people over 85-years old.^15^ Our study found that the risk for indwelling catheterization rises with age as well, especially for men. A total of 0.8% of 65–75-year-old men used at least one IC in 2018. In the same year, an IC was used in 8.1% of males above 85-years old. A similar increase was seen for CIC use in older patients. It confirms that the need for urinary drainage increases with age and male sex, especially for indwelling catheterization.

This study is the first describing the prevalence of neurogenic and non-neurogenic patients with urinary catheters in a total population. Small studies have been performed to address this in specific neurogenic bladder patient groups, but precise numbers could not be estimated.^4^ We described the amount of catheter users with neurogenic and non-neurogenic causes in 2018. Of all users, 20.7% had a neurogenic cause as underlying disease. Almost half of those users only applied indwelling catheterization. This relatively high number might be explained by urge incontinence in neurogenic bladder patients or impaired hand function in neurogenic patients. For a total of 34.4% all urinary catheter users, no underlying cause was distinguished due to unknown DBC codes. Individuals started before 2012, without control visits in the hospital, lacked a registered DBC code, and therefore, could not be classified into a neurogenic or non-neurogenic cause. A precise distribution for neurogenic and non-neurogenic patients could therefore not be made, resulting in a possible underestimation for the neurogenic bladder population. It is unknown if DBCs are registered after a urodynamic investigation is conducted; therefore, caution is necessary for the interpretation of all registered DBCs as to the underlying disorder.

The costs of outpatient medical devices in the Netherlands are rising, and urinary catheters are in the top four of highest expenditures.^16^ Generalized to the global market size for urinary catheters, estimated at 3.4 billion dollars in 2015, the Netherlands contributes up to 2.6% of the expenditures for urinary catheters worldwide.^8^ In 2018, 84% (75.5 million euros) of the total expenditure on extramural urinary catheters and urine drainage bags was for CIC use.

It should be noted that these results are limited to the Dutch setting. In the Netherlands, and most European Union countries, most catheters are reimbursed by healthcare insurance. Unfortunately, medical insurance coverage levels tend to be lower, especially in low-income countries. Socioeconomic aspects, such as the price of the catheter or the number of catheters needed in a specific time period and healthcare standards, are factors that may influence the choice of catheter type. Therefore, this study may represent a biased ‘optimal situation’ for countries without full insurance coverage.

Our results are in line with the IC use in England, Wales, and Northern Ireland. ICs were present in 3% of the people living in the community, as is in our study. An IC was used in 13% of care home residents.^17^ We suspect that the use of indwelling and external catheters is also substantially higher in the Dutch care facilities (intramural). Although we put much effort into finding a suitable database on intramural use, this study only contains information about the extramural (outpatient) use of urinary catheters. Therefore, the main limitation of this study is that the use of urinary catheters in hospitals and care centers (e.g. rehabilitation centers) are not included in this analysis. This might have resulted in an underestimation, especially for indwelling and external urinary catheters. However, the data on extramural use is unique, as it is the first study using a population-based cohort.

Although the current study is based on the total non-hospitalized and non-institutionalized Dutch population, the findings show a substantial growth over the past 2 decades in use and costs for indwelling and intermittent urinary catheters. One fifth of all users had a neurogenic underlying condition, and almost half of those patients used only indwelling catheterization, which has never been described before. Only a part of the increased costs for catheters was due to price increase from manufacturers, while urine-drainage bag prices decreased, evidently. It seems that the main driver behind the prevalence in IC and CIC use is the rise in incontinence in older patients and the adaption of preferred CIC use in professional guidelines.

## Supplemental Material

sj-xlsx-1-tau-10.1177_17562872211007625 – Supplemental material for Urinary catheterization from 1997 to 2018: a Dutch population-based cohort Click here for additional data file. Supplemental material, sj-xlsx-1-tau-10.1177_17562872211007625 for Urinary catheterization from 1997 to 2018: a Dutch population-based cohort by Sophie A. Berendsen, Tess van Doorn and Bertil F. M. Blok in Therapeutic Advances in Urology
